# New Alzheimer’s disease diagnostics and psychological clinical perspectives

**DOI:** 10.3389/fpsyg.2026.1866853

**Published:** 2026-09-17

**Authors:** Maria Tereza Mendes Aversi, Patricia Martins Freitas, Tales Alexandre Aversi-Ferreira

**Affiliations:** 1 Postgraduate Program in Physiopathology and Medical Clinics, School of Medicine, São Paulo State University (UNESP), Botucatu, Brazil; 2 School of Psychology, Federal University of Bahia (UFBA), Vitória da Conquista, Brazil; 3 Biomathematics Laboratory, Institute of Sciences and Technology (ICT), Federal University of Alfenas (UNIFAL-MG), Poços de Caldas, Brazil

**Keywords:** Alzheimer’s disease, blood-based biomarkers, cognitive rehabilitation, dementia prevention, multidisciplinary care, psychological intervention

## Abstract

Recent advances in blood-based biomarkers have transformed the early diagnosis of Alzheimer’s disease (AD), enabling the identification of biological risk at preclinical and presymptomatic stages in a noninvasive and accessible manner. Despite this diagnostic progress, the expansion of psychological clinical services in dementia care has not kept pace, representing a critical gap in the current management of AD. This theoretical paper critically discusses the role of psychological clinical practice in the prevention and management of AD in light of these diagnostic advances, particularly from the perspective of the International Working Group (IWG), which defines AD as a clinical-biological entity and recommends that cognitively normal individuals with positive biomarkers be considered at risk rather than diagnosed. Based on this framework and on evidence from the 2024 Lancet Commission on dementia prevention, we propose a three-phase roadmap for the integration of psychological care into multidisciplinary AD management. Phase 1 encompasses universal prevention through psychoeducation, cognitive reserve promotion, and emotional education. Phase 2 targets at-risk individuals with elevated biomarker profiles, focusing on neuropsychological evaluation, risk disclosure support, and early cognitive intervention. Phase 3 addresses individuals with established AD through cognitive rehabilitation and caregiver support. We further argue that government policies must integrate psychologists into multidisciplinary neurological and geriatric teams, and that psychology training programs must be updated to prepare professionals for preventive roles. More research on standardized psychological protocols for pre-dementia contexts is urgently needed.

## 1 Introduction

Alzheimer’s disease (AD) was first described by the German psychiatrist Alois Alzheimer in 1906, following his neuropathological examination of patient Auguste Deter, in which he identified neuritic (senile) plaques—later shown to contain amyloid-β—and neurofibrillary tangles as hallmarks of the disease (Bermejo-Pareja and Del Ser, 2024; Trevisan et al., 2019). More than a century later, its etiology remains complex and not fully elucidated, involving a multifactorial interplay of genetic factors, particularly the APOE ε4 allele and mutations in APP, PSEN1, and PSEN2 genes, and environmental risk factors, including air pollution, diet, metabolic disorders, and psychosocial stress (Mertaş and Boşgelmez, 2025; Safiri et al., 2024).

AD is strongly associated with aging (Trevisan et al., 2019), and its prevalence increases steeply after the age of 65, rising from approximately 5% in individuals aged 65–74 to 13% in those aged 75–84 and more than 33% in those aged 85 or older (Alzheimer’s Association, 2024). In the United States alone, an estimated 6.9 million people aged 65 and older were living with Alzheimer’s dementia in 2024 (Alzheimer’s Association, 2024), while globally, more than 55 million people were living with dementia in 2020, a figure projected to reach 78 million by 2030 and to nearly double again to 139 million by 2050, with the fastest growth occurring in low- and middle-income countries (Alzheimer’s Disease International, 2023).

This age-dependent and exponential growth generates a substantial burden on both populations and healthcare systems, with total care costs in the United States alone estimated at US$ 360 billion in 2024 and projected to reach nearly US$ 1 trillion by 2050 (Alzheimer’s Association, 2024; Tay et al., 2024). Beyond direct healthcare expenditure, this burden falls disproportionately on unpaid family caregivers, whose emotional, physical, and financial strain is increasingly recognized as a public health concern in its own right and a determinant of care quality and sustainability (Tay et al., 2024).

Over the past two decades, the diagnosis of AD has undergone substantial transformations, shifting from a purely clinical and syndromic approach to a biologically based framework grounded in objective biomarker evidence (Khalafi et al., 2025; Medina-Rioja et al., 2026). This paradigm shift was driven by advances in neuroimaging, particularly amyloid and tau positron emission tomography (PET), and by the development and validation of cerebrospinal fluid (CSF) biomarkers, culminating most recently in the emergence of highly accurate blood-based biomarkers (BBMs), especially plasma phosphorylated tau 217 (p-tau217) (Jack et al., 2018, 2024; Khalafi et al., 2025; Ottoy et al., 2025; Roh et al., 2026).

Unlike PET imaging and CSF sampling, which require specialized infrastructure, radiotracers, or lumbar puncture and are therefore largely confined to tertiary academic centers, BBMs can be obtained through a routine venous blood draw, making large-scale, population-level screening for AD pathology logistically and economically feasible for the first time (Hampel et al., 2023; Khalafi et al., 2025). Plasma p-tau217 in particular has emerged as the analyte of choice, achieving a level of diagnostic accuracy previously attainable only through PET or CSF analysis (Khalafi et al., 2025; Medina-Rioja et al., 2026). The 2018 National Institute on Aging–Alzheimer’s Association (NIA-AA) Research Framework first formally defined AD as a biological, rather than syndromic, construct based on the amyloid/tau/neurodegeneration [AT(N)] classification, while the 2024 Revised Criteria for Diagnosis and Staging incorporated plasma biomarkers as Core 1 diagnostic tools, enabling earlier and less invasive identification of AD pathology across its full continuum (Jack et al., 2024; Kobayashi et al., 2026).

While greater diagnostic precision is expected to enable more targeted and timely interventions, it is increasingly recognized that lifelong prevention represents the most effective strategy to reduce the burden of AD (Alzheimer’s Disease International, 2023; Livingston et al., 2024; Trevisan et al., 2019). Psychological clinical practice plays an essential role in this prevention-oriented approach, particularly through the management of modifiable risk factors—such as depression, social isolation, chronic stress, and cognitive inactivity—that are amenable to psychological intervention throughout the life course (Graham-Engeland et al., 2025; Livingston et al., 2024).

Indeed, the 2024 Lancet Commission estimated that addressing 14 modifiable risk factors across the lifespan—several of which are psychological or behavioral in nature, including depression, social isolation, and cognitive inactivity—could potentially prevent or delay up to 45% of dementia cases worldwide (Livingston et al., 2024), underscoring psychology’s centrality to any credible prevention strategy rather than a peripheral or purely supportive role.

Moreover, the shift toward biomarker-based risk stratification introduces a distinctly psychological challenge that did not exist under the earlier symptomatic diagnostic paradigm: cognitively healthy individuals can now be informed that they carry a positive biological substrate for AD years or even decades before any clinical symptoms might emerge, with profound implications for identity, anxiety, and family dynamics that purely medical or biological expertise is not equipped to address (Dubois et al., 2024).

Nevertheless, despite the remarkable advances in biological diagnosis over the past decade, these developments have not been accompanied by a proportionate expansion of psychological clinical services in dementia care, representing a critical and largely unaddressed gap in the current management of AD (Roh et al., 2026; Safiri et al., 2024).

In this context, it is reasonable to hypothesize whether psychological clinical interventions could prevent or delay the manifestation of AD. This manuscript is conceived as a Perspective (Hypothesis and Theory) article: its purpose is not to exhaustively catalog the existing literature, but to critically articulate a novel theoretical framework connecting recent advances in blood-based biomarkers to psychological clinical practice.

Accordingly, the aim of this theoretical paper is to present and critically discuss the role of psychological clinical practice in the management and prevention of AD, in light of the recent diagnostic advances that enable the identification of biological risk at preclinical stages. Specifically, drawing on the International Working Group’s (IWG) clinical-biological framework and recent evidence on modifiable risk factors, we propose and justify a three-phase roadmap—spanning universal prevention, targeted intervention for biomarker-defined at-risk individuals, and support for those with established disease—for integrating psychological clinical practice into multidisciplinary AD prevention and management, together with the professional, ethical, and policy conditions required for its implementation.

## 2 Search strategy and methodology

This manuscript adopts a narrative, theory-driven literature synthesis appropriate to a Perspective (Hypothesis and Theory) article, rather than a systematic review. The literature search was conducted in PubMed/MEDLINE, Scopus, Web of Science, and Google Scholar, using combinations of the keywords ‘Alzheimer’s disease’, ‘blood-based biomarkers’, ‘p-tau217’, ‘diagnostic criteria’, ‘International Working Group’, ‘NIA-AA’, ‘psychological intervention’, ‘dementia prevention’, ‘modifiable risk factors’, ‘caregiver’, and ‘multidisciplinary care’. This approach follows the narrative synthesis strategy previously employed (Trevisan et al., 2019), complemented by a focused search of recent clinical and biomarker literature, following the model of recent opinion-based analyses in this field (Medina-Rioja et al., 2026).

No restriction was applied regarding study design: original research, systematic reviews, meta-analyses, opinion and perspective articles, clinical guidelines, and classic theoretical works were all eligible. Regarding publication date, the search prioritized literature published between 2011 and 2026, reflecting the period in which blood-based biomarkers and the revised NIA-AA/IWG diagnostic frameworks emerged and rapidly evolved; foundational theoretical and methodological references predating this window (e.g., Vygotsky, 1978; Clare and Woods, 2004; Strauss et al., 2006; Green et al., 2009) were retained when directly relevant to the conceptual framework proposed. Only articles published in English, Portuguese, or Spanish were considered, and sources falling outside the intersection of AD diagnostics, prevention, and psychological clinical practice were excluded.

## 3 Diagnostic advances and paradigm shift in Alzheimer’s disease

Prior to the current biological diagnostic era, the clinical diagnosis of AD relied primarily on the identification of characteristic symptoms—such as progressive cognitive decline, impaired acquisition of new information, and visuospatial disorientation interfering with daily functioning—combined with neuropsychological testing and the exclusion of other dementia subtypes (Frisoni et al., 2025; Safiri et al., 2024). Subsequently, advances in neuroimaging techniques and CSF analysis, particularly through the detection of tau protein deposits within neurofibrillary tangles and amyloid-β plaques, enabled more reliable *in vivo* diagnostic approximations (Baviskar and Mahajan, 2026; Mu et al., 2024). Nevertheless, prior to the advent of validated biomarkers, post-mortem neuropathological examination remained the only method by which a definitive diagnosis could be established with certainty (Frisoni et al., 2025; Medina-Rioja et al., 2026).

Among the blood-based biomarkers currently available, analytical performance varies considerably, which directly shapes their clinical utility across the AD continuum. Plasma p-tau217 shows the strongest evidence base, with pooled sensitivity and specificity of approximately 82–86% for detecting amyloid and tau pathology in meta-analysis (Khalafi et al., 2025), and estimates approaching 90% in the largest synthesis to date (Therriault et al., 2025). Its ratio with Aβ42 became the first blood-based assay cleared for diagnostic use, making it the analyte of choice for confirming AD pathology; it is important to note, however, that this clearance covers adults aged 55 years and older who already present signs and symptoms of cognitive decline, and explicitly excludes stand-alone screening use, so biomarker testing of cognitively unimpaired individuals as proposed in Phase 2 remains a research rather than an approved clinical indication (Khalafi et al., 2025; Medina-Rioja et al., 2026). Plasma p-tau181, an earlier-generation marker, offers adequate but comparatively lower diagnostic accuracy and is more susceptible to confounding by renal function and body mass index (Medina-Rioja et al., 2026).

Neurofilament light chain (NfL), while not specific to AD, is a sensitive marker of axonal injury and neurodegeneration more broadly, useful for monitoring disease progression and for distinguishing neurodegenerative from primary psychiatric and other non-neurodegenerative conditions, rather than for initial diagnosis of AD (Hampel et al., 2023). The plasma Aβ42/Aβ40 ratio, although the earliest blood-based marker developed, shows only modest discriminative accuracy and is typically combined with p-tau217 to increase diagnostic confidence (Hampel et al., 2023; Khalafi et al., 2025).

From a clinical-psychological standpoint, this differentiated analytical performance has direct implications for when, in Phase 2, the psychologist’s involvement should begin. Because plasma p-tau217 offers the strongest positive and negative predictive value, a positive p-tau217 result, particularly when confirmed by an elevated p-tau217/Aβ42 ratio, should trigger immediate psychological engagement, given the higher probability that the result reflects true underlying AD pathology and, consequently, a proportionally greater risk of iatrogenic distress upon disclosure. In contrast, isolated elevations in less specific markers, such as NfL, warrant a more cautious approach, in which psychological support is offered alongside diagnostic clarification rather than as a direct response to an at-risk label, avoiding premature psychological burden associated with results of uncertain interpretive value (Hampel et al., 2023; Ottoy et al., 2025).

Recent diagnostic criteria introduced two major novelties: (a) the addition of a preclinical and asymptomatic stage that can be detected in individuals without symptoms, and (b) the incorporation of biological aspects into the AD diagnosis (Jack et al., 2024). In contrast, another group defended a clinical-biological approach that links clinical features with biomarker detection, arguing that individuals with positive biomarkers should be considered at risk but should not receive a formal diagnosis of AD according to the International Working Group (IWG) approach (Dubois et al., 2024).

In fact, these two frameworks reflect a genuine conceptual dilemma rather than a mere terminological difference. The 2024 NIA-AA Revised Criteria formally designate biomarker-positive, cognitively unimpaired individuals as being in an early biological stage of AD (Stage 1 of the 2024 clinical staging scheme), treating biomarker positivity itself as sufficient grounds for diagnosis, independent of clinical symptoms (Jack et al., 2024).

The IWG, by contrast, requires the co-occurrence of a compatible clinical phenotype and positive biomarkers for a formal AD diagnosis, reserving the label ‘at risk’ for biomarker-positive individuals who remain cognitively normal (Dubois et al., 2024). This distinction is not merely semantic: it determines whether a person who may never develop clinical symptoms is told they ‘have’ a fatal neurodegenerative disease. Given that a substantial proportion of biomarker-positive, cognitively unimpaired individuals do not progress to symptomatic AD within observable timeframes, and considering that, although the average psychological impact of risk disclosure appears low, a clinically meaningful minority of recipients experience anxiety, depressive symptoms, or stigmatization—and that the available evidence derives largely from genetic rather than plasma-biomarker disclosure in selected research volunteers (Green et al., 2009)— the IWG’s more conservative, risk-based terminology is ethically preferable from a psychological standpoint: it preserves diagnostic caution, avoids premature identity disruption, and creates the discursive space needed for structured, preventive psychological intervention, rather than crisis-oriented management of a disease label, during Phase 2 of the proposed roadmap in this work.

The IWG approach helps to avoid labeling cognitively healthy individuals with a disease diagnosis when they may never develop AD, thereby preventing undesirable psychological and social consequences (Dubois et al., 2024). However, early and careful identification of risk opens a significant opportunity for psychological clinical work to support individuals classified as “at risk,” both those with elevated biomarker-based risk and those in the presymptomatic stage (Dubois et al., 2024; Rostamzadeh et al., 2022).

This represents a therapeutic window for psychological management aimed at preventing maladaptive behaviors, such as depression, anxiety, social isolation, and chronic stress, that may contribute to AD onset or progression, given that diagnosis should be offered only when clinically indicated (Graham-Engeland et al., 2025; Livingston et al., 2024; Rostamzadeh et al., 2022). In this context, the presence and active role of psychologists in multidisciplinary teams is essential and has direct implications for public health policy regarding the profession (Dandan et al., 2024; Dubois et al., 2024).

## 4 Psychological approaches to managing modifiable risk factors

According to Livingston et al. (2024), 14 risk factors for AD are modifiable, namely, low educational attainment, hearing loss, hypertension, smoking, obesity, depression, physical inactivity, diabetes, excessive alcohol consumption, traumatic brain injury, air pollution, social isolation, high LDL cholesterol (in midlife), and untreated visual impairment (in later life). Evidence-based psychological practice offers significant contributions across all these domains. Specifically: for depression, through psychotherapy, particularly cognitive behavioral therapy (CBT) and behavioral therapy (Livingston et al., 2024; Rostamzadeh et al., 2022); for social isolation, through community support and social engagement interventions (Livingston et al., 2024); for physical inactivity, through motivational and behavioral psychology (Livingston et al., 2024); for smoking cessation and excessive alcohol consumption, through behavioral interventions and harm reduction strategies (Livingston et al., 2024); for chronic stress, through mindfulness-based approaches, emotional regulation techniques, and CBT (Graham-Engeland et al., 2025); and for the cognitive vulnerability associated with limited educational attainment, through cognitive stimulation and cognitive reserve enhancement (Livingston et al., 2024; Rostamzadeh et al., 2022).

However, the limitations of psychological intervention must be acknowledged: several of the modifiable risk factors—such as hypertension, diabetes, LDL cholesterol, hearing loss, air pollution, traumatic brain injury, and untreated visual impairment—are primarily addressed through pharmacological, surgical, audiological, or environmental and regulatory measures, with psychology contributing only to specific behavioral and adherence-related aspects (Livingston et al., 2024; Park et al., 2024).

Presenting more limited randomized evidence but expanding the scope of psychologist actions, psychoanalytic and psychodynamic approaches offer meaningful contributions to the management of depression, chronic stress, and social isolation in older adults, particularly through the exploration of unconscious processes, intrapsychic conflicts, and early life trauma that may underlie maladaptive behaviors and emotional suffering (Ambresin et al., 2023; Seiler et al., 2025). Psychodynamic and group analytic approaches have shown evidence of reducing emotional distress and social isolation in elderly populations, addressing existential dimensions of aging that behavioral therapies may not fully encompass (Seiler et al., 2025).

From a socio-historical psychology perspective grounded in Vygotsky’s (1978) cultural-historical framework, cognitive development and higher psychological functions are understood as shaped by social interaction, cultural tools, and historical context. This perspective supports interventions aimed at enhancing cognitive reserve through meaningful social and cultural participation, addressing low educational attainment through mediated learning strategies, and framing dementia prevention as a collective and public health endeavor embedded in social and institutional structures (Livingston et al., 2024; Vygotsky, 1978).

Regardless of the approach employed, psychology serves as a critical link between the modifiable risk factors for AD, as identified considering recent diagnostic advances, and preventive clinical intervention (Dubois et al., 2024; Livingston et al., 2024; Rostamzadeh et al., 2022).

It is important to conceptually distinguish, throughout this proposal, between psychological intervention in the broad sense, encompassing psychotherapeutic approaches such as CBT, psychodynamic therapy, and socio-historically grounded interventions aimed at modifiable risk factors and emotional well-being, as discussed above, and neuropsychological practice, a specialized subfield concerned with the assessment and rehabilitation of cognitive functions (memory, attention, executive function, language) through standardized, norm-referenced instruments and structured cognitive training protocols (Alzola et al., 2024; Lezak et al., 2012).

While both domains fall under psychology and are frequently performed by the same professional in contexts where postgraduate specialization in neuropsychology is standard, they serve complementary but distinct functions across the proposed roadmap: neuropsychological assessment is central to detecting and characterizing early cognitive change (Phase 2) and monitoring functional decline (Phase 3), whereas psychotherapeutic and psychoeducational interventions primarily target the emotional, behavioral, and social dimensions of risk reduction and adaptation across all three phases. Recognizing this distinction is essential for workforce planning and training policy, since neuropsychological assessment requires specific technical certification beyond general clinical psychology training (Lezak et al., 2012).

## 5 Clinical, ethical, and professional development challenges

Several challenges are associated with the work of psychologists in the pre-dementia clinical field. A particularly complex issue concerns the communication of risk status to patients—that is, informing them that they are at increased risk for AD—and the equally important shared decision-making process involving the family and other healthcare professionals regarding whether and how such disclosure should be made (Alpinar-Sencan and Schicktanz, 2020; Dubois et al., 2024; Rostamzadeh et al., 2022).

Communicating the risk of AD can trigger negative emotional responses, such as anxiety and depression, increasing the risk of developing the disease. To safely control this effect, preventive work must be systematic—that is, structured, protocol-based, and continuous rather than occasional, focusing on reducing risk factors, increasing protective factors, and establishing a set of strategies that meet the quality-of-life demands of the person at risk and their family members (Heilman and Nadeau, 2022; Ismail et al., 2018).

This risk of iatrogenic harm warrants more direct and sustained attention: although randomized and observational studies of risk disclosure have generally found no significant average increase in anxiety or depression (Green et al., 2009), a clinically meaningful minority of recipients do experience iatrogenic anxiety, depressive symptoms, or a lasting sense of stigmatization, even when the person may never progress to clinical AD (Erickson et al., 2021). Because this evidence base derives predominantly from genetic risk disclosure in selected, highly educated research cohorts, its generalizability to routine plasma-biomarker disclosure in the general population remains uncertain, which is precisely why psychological monitoring should be built into the process rather than assumed to be unnecessary. Because biomarker positivity does not equate to a certain diagnosis, psychologists must be actively involved throughout the disclosure process, not only in its immediate aftermath, but providing structured pre-test counseling, careful communication of probabilistic risk, and continuous emotional support to prevent premature identity changes, social withdrawal, or discrimination linked to an ‘at-risk’ label (Alpinar-Sencan and Schicktanz, 2020; Dubois et al., 2024; Erickson et al., 2021). Ethical guidelines for risk disclosure should explicitly address these psychosocial harms as a primary clinical concern, on equal footing with the accuracy of the biomarker result itself, given that inadequately managed disclosure can, paradoxically, generate greater psychological morbidity than the biological risk it communicates.

Building on this need for structured disclosure, we further argue that informed consent for blood-based biomarker testing should not be treated as a single, one-time signature obtained at sample collection, but rather as a dynamic, staged process revisited at each critical decision point. Concretely, this entails at least three distinct moments of consent, i.e., (1) consent to undergo testing itself, preceded by pre-test counseling that clarifies the probabilistic and non-diagnostic nature of a positive result, the possibility of false positives, and the psychological implications of knowing one’s biological status (Green et al., 2009); (2) an explicit, separate consent to actually receive the result, since patients should retain the right to decline disclosure even after agreeing to be tested; and (3) ongoing consent to ancillary uses of the information, such as sharing results with family members or other care providers. Embedding psychologists in this consent process, rather than treating consent as an exclusively medical or administrative formality, ensures that the emotional readiness of the patient, not only their biological eligibility, guides the decision to learn a biomarker result (Erickson et al., 2021).

The expectation is that, by addressing potential barriers in the healthcare field and the diverse services offered in each country, preventive mechanisms are integrated into a primary care model, thereby significantly reducing the likelihood of disease manifestation. In general, this represents an improvement in the health of the at-risk population. On the other hand, it represents a mechanism with economic implications, as the non-manifestation of AD reduces costs for public health services (Schwarz et al., 2022).

Significant gaps also remain in psychologist training for pre-dementia preventive contexts across many training programs worldwide, particularly in low- and middle-income countries (LMICs; Kalaria et al., 2024; Livingston et al., 2024). These gaps are compounded by resistance within many health systems to fully incorporating psychologists into multidisciplinary care teams, particularly in neurology and geriatrics (Dandan et al., 2024). Additionally, it is imperative to develop standardized clinical protocols for preventive psychological services in the pre-dementia context, given the current absence of consensus guidelines specifically addressing the psychological management of individuals at risk for AD (Atri et al., 2025; Dickerson et al., 2025; Rostamzadeh et al., 2022). Atri et al. (2025) and Dickerson et al. (2025) are companion executive summaries of the same Alzheimer’s Association DETeCD-ADRD guideline, addressed, respectively, to primary and specialty care settings; neither provides standardized protocols for psychological intervention, underscoring the gap identified here.

## 6 Proposed roadmap for psychological action in AD prevention

Considering the possibilities of early diagnosis of AD provided by recent blood-based biomarkers, which permit a noninvasive approach (Liu et al., 2024a), and the theory that AD is a consequence of aging, probably a manifestation of the normal decline of cellular metabolism (Trevisan et al., 2019), preventive care directed at the general population represents the most effective approach to avoiding AD and dementia in general. Blood-based biomarkers may enable earlier and faster diagnoses and aid in risk assessment, early detection, prognosis, and management (Hampel et al., 2023). A community-based cohort study found that blood-based biomarkers for AD could predict dementia up to 16 years before disease onset (Grande et al., 2025).

In this context, we propose a three-phase roadmap for integrating psychological clinical practice into AD prevention and management.

### 6.1 Phase 1—universal prevention

Programs of psychoeducation, cognitive reserve promotion, management of depression and stress, and emotional education in schools (Goleman, 2012) could be implemented as general actions by psychologists to prevent AD and cognitive senescence from early life onward. The 2024 Lancet Commission on dementia provides hopeful evidence about dementia prevention, intervention, and care (Livingston et al., 2024). Cognitive reserve across different life stages has been associated with a reduced risk of dementia (Liu et al., 2024b), and non-pharmacological dementia risk-reduction interventions typically target multiple risk factors—such as cardiovascular health, diet, physical activity, and cognitive and social engagement—through psychoeducation and behavior-change techniques (Amos et al., 2024).

Telepsychology and other digital psychological tools offer a particularly cost-effective and scalable mechanism for operationalizing Phase 1 at a population level, enabling remote delivery of psychoeducation, emotional-regulation training, and cognitive-reserve-promoting activities through mobile applications, web-based platforms, and videoconference-delivered group sessions, thereby overcoming geographic and workforce-related access barriers (Amos et al., 2024). In Phase 2 (see below), digital tools additionally support large-scale monitoring of at-risk individuals between in-person neuropsychological evaluations, through remote cognitive testing, ecological momentary assessment of mood and daily functioning, and telehealth-delivered follow-up counseling after risk disclosure, extending the reach of specialized psychological services to underserved and remote populations (Xu and Fu, 2025). Nonetheless, the adoption of these tools should be considered as complementary to, rather than a substitute for, in-person care in cases involving high emotional vulnerability or complex diagnostic communication, and their implementation must account for the digital literacy and connectivity constraints discussed below in relation to cultural and socioeconomic diversity (see Figure 1).

**Figure 1:**
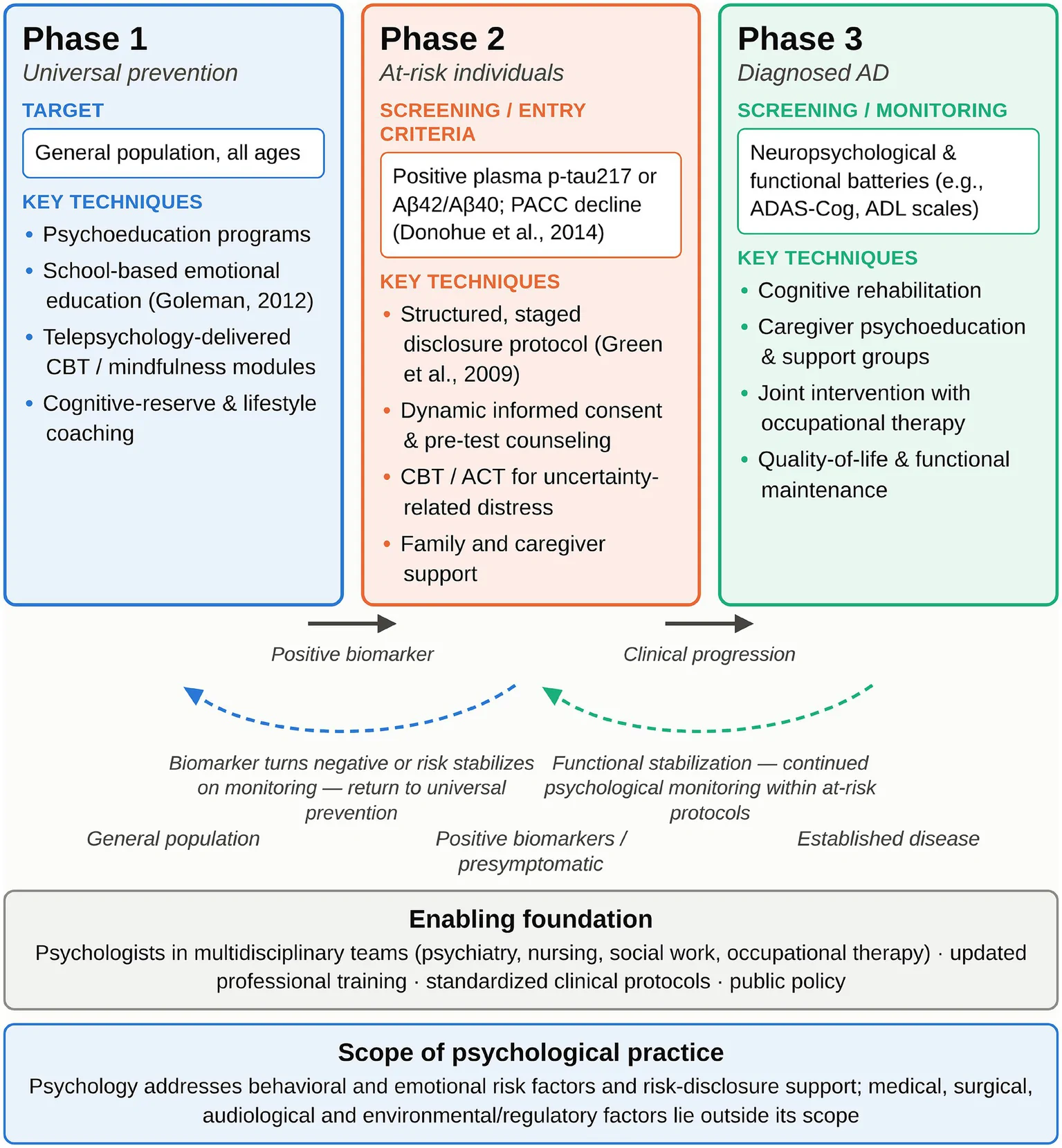
Three-phase roadmap for integrating psychological care into Alzheimer’s disease prevention and management, aligned with the IWG at-risk framework and the 2024 Lancet Commission. Phase 1 (universal prevention) targets the general population; Phase 2 (targeted prevention) targets at-risk individuals meeting blood-based biomarker screening criteria; Phase 3 addresses individuals with diagnosed AD. Each box lists the screening/entry criteria and the key psychological techniques recommended for that phase. Dashed arrows indicate feedback loops for cases in which biomarker or functional status stabilizes, returning the individual to a less intensive tier of monitoring rather than automatic advancement to the next phase. The lower bands summarize the structural conditions required for implementation (enabling foundation) and the scope of psychological practice.

### 6.2 Phase 2—targeted prevention for at-risk individuals

This phase focuses on individuals at elevated risk of developing AD according to biological tests such as blood-based biomarkers and clinical observations, using neuropsychological evaluation, emotional support following risk disclosure, and cognitive intervention. Neuropsychological assessment plays a fundamental role in characterizing cognitive decline at different stages of dementia, enabling early detection and the differential diagnosis of neurodegenerative disorders (Alzola et al., 2024). These procedures aim to support both the patient and the family, considering that the individual may not develop AD, while also promoting psychological education to help manage the impact of an early-risk identification. Recent clinical practice guidelines emphasize the importance of a structured counseling and disclosure process, underscoring that the involvement of a care partner throughout is essential, since cognitive symptoms may compromise a person’s ability to process complex diagnostic information independently (Atri et al., 2025). If health professionals and the family decide to disclose the risk status to the patient, bearing in mind that the presence of a positive biological substrate for AD does not inevitably mean that the individual will manifest the disease, psychological support must be provided, at minimum, to the family.

In practical terms, Phase 2 implementation in primary or specialized care settings should combine sensitive, standardized neuropsychological instruments capable of detecting subtle, amyloid-related cognitive decline in preclinical and prodromal stages, such as the Preclinical Alzheimer Cognitive Composite (PACC), which integrates episodic memory, processing speed, and global cognition measures specifically calibrated to track early biomarker-associated decline (Donohue et al., 2014), with structured, manualized psychological support protocols for risk communication.

Disclosure sessions should follow a stepwise, counselor-assisted model, informed by structured protocols originally developed for genetic risk disclosure (e.g., the REVEAL study) and adaptable to blood-based biomarker results, comprising pre-test counseling, in-person result disclosure with a trained professional, and scheduled follow-up sessions to monitor emotional adjustment (Green et al., 2009). Complementing disclosure support, brief manualized interventions such as psychoeducation modules on modifiable risk factors, structured cognitive stimulation programs (Lu et al., 2025), and short-term cognitive-behavioral or acceptance-based protocols targeting uncertainty-related distress (Saragih et al., 2026; Zhou et al., 2025) should be offered to at-risk individuals as first-line, low-intensity psychological care, with referral to more intensive psychotherapy reserved for those presenting clinically significant anxiety or depressive symptoms following disclosure.

The psychological impact of biomarker-based risk identification extends well beyond the individual who receives the result. Family members and prospective caregivers of at-risk individuals frequently experience significant emotional overload, anticipatory grief, and chronic uncertainty long before any clinical symptoms emerge, as they begin to reorganize future expectations, caregiving roles, and family dynamics around a diagnosis that may never materialize (Alpinar-Sencan and Schicktanz, 2020; Livingston et al., 2024).

This anticipatory emotional burden, sometimes described as pre-caregiving stress, can generate its own psychological morbidity, including heightened anxiety, hypervigilance toward the at-risk person’s cognitive performance, and strain within couple and family relationships, independently of whether the at-risk individual ultimately progresses to AD (Rostamzadeh et al., 2022). Accordingly, Phase 2 psychological support should be explicitly extended to caregivers and family members as co-recipients of care, rather than as secondary beneficiaries, through dedicated psychoeducation on the probabilistic nature of biomarker risk, structured spaces for processing anticipatory grief, and early guidance on sustainable caregiving practices that can be built upon should the disease eventually manifest.

#### 6.2.1 Phase 3—intervention for diagnosed AD

Following the onset of AD in its initial stages, Phase 3 requires the psychologist’s work to become more detailed and to demand greater professional expertise, particularly for implementing cognitive rehabilitation and organizing support for caregivers. Cognitive rehabilitation for people living with dementia is a behavioral therapy focused on managing the impact of cognitive impairment on everyday life; it does not aim to cure dementia but to enable the optimal level of functioning despite the condition, with the ultimate goal of improving quality of life and well-being (Fondation Médéric Alzheimer, 2024). This is the type of action psychologists most commonly perform at present, given that the history of AD has long been marked by the difficulty of providing an early diagnosis until recent years.

The feasibility of this roadmap (Figure 1) is not uniform across the globe: access to blood-based biomarker testing and to specialized psychological care remains markedly unequal between high-income countries and LMICs, where diagnostic infrastructure, laboratory capacity, and reimbursement mechanisms for novel biomarkers are considerably less developed (Kalaria et al., 2024). Cultural factors further shape how risk information is received and how psychological interventions should be delivered, as beliefs about aging and dementia, family-centered versus individual-centered decision-making, language barriers, and differing levels of health literacy all influence the acceptability and effectiveness of psychoeducation, risk disclosure, and cognitive intervention programs (Livingston et al., 2024). Consequently, the phases proposed here should not be implemented as a uniform template; rather, Phase 1 psychoeducational content, Phase 2 disclosure protocols, and Phase 3 caregiver support strategies must be culturally adapted and co-developed with local communities and health systems, particularly in vulnerable socioeconomic contexts where mistrust of biomedical institutions, stigma surrounding cognitive decline, or limited access to digital health infrastructure may otherwise undermine adoption (Kalaria et al., 2024; Livingston et al., 2024).

Translating the proposed phases into measurable practice requires governmental commitment, particularly through the formal hiring of psychologists in health centers and their structured incorporation into multidisciplinary teams in neurological and geriatric services. Embedding psychologists in both public and private healthcare systems is pivotal for ensuring comprehensive, equitable, and humanized care across the full spectrum of clinical complexity. At the primary care level, psychological practice is oriented toward health promotion, early identification of psychological distress, psychoeducation, and matrix support to Family Health Teams, thereby strengthening the resolutive capacity of the Unified Health System [in Portuguese, “Sistema Único de Saúde” (SUS)] in Brazil, for instance, and reducing the systemic burden generated by unmet mental health demands (Alves et al., 2024; Braga and Borges, 2025). At the secondary care level, psychologists contribute to diagnostic precision, specialized neuropsychological assessment, and consultation–liaison practices that support the management of patients with complex medical–psychiatric comorbidities—an integration consistently associated with shorter hospital stays, improved adherence, and enhanced quality of care (Müller and Ortega, 2024; Tan et al., 2024). At the tertiary care level, the role of psychologists becomes particularly critical in rehabilitation psychology and in the longitudinal management of chronic, disabling, and neurodegenerative conditions, where multimodal psychological interventions optimize functional recovery, emotional adjustment, and caregiver support (Bates et al., 2025; Xu and Fu, 2025).

Beyond improving clinical endpoints, this multiprofessional integration consolidates the humanization, sustainability, and person-centeredness of contemporary healthcare systems, aligning psychological science with the ethical and political commitments of universal health coverage (Shore et al., 2025).

In this way, collaborative dementia care models address dementia-related medical and psychosocial needs through interdisciplinary teams that extend the reach of the limited dementia specialty workforce, coordinate care, and improve quality of life for both the patient and the caregiver (Possin et al., 2024). Person-centered, multidimensional, and interdisciplinary rehabilitation can increase the opportunities for older adults with dementia and their informal primary caregivers to continue to live an active life and participate in society (Lindelöf et al., 2023).

In fact, the psychologist’s role within this interdisciplinary team extends well beyond geriatrics and neurology. Close operational interaction with psychiatry is essential for the pharmacological management of comorbid mood, anxiety, and behavioral disturbances that frequently accompany both the at-risk state and established AD, requiring joint case formulation and shared monitoring of treatment response (Dandan et al., 2024). Collaboration with skilled nursing staff supports the continuous, day-to-day observation of functional and behavioral changes that inform the timing and intensity of psychological intervention, particularly in institutional and home-care settings. Partnership with social workers is critical for addressing the socioeconomic determinants of access discussed above, such as transportation, caregiver financial strain, and eligibility for social benefits, that directly affect adherence to psychological and medical follow-up. Finally, joint work with occupational therapists strengthens Phase 3 interventions by translating cognitive and emotional gains from psychotherapy and neuropsychological rehabilitation into concrete improvements in activities of daily living and functional independence (Possin et al., 2024). Formalizing these interfaces, through joint case conferences, shared care plans, and clearly defined referral pathways, is a precondition for the roadmap proposed here to function as an integrated system rather than a set of parallel, uncoordinated services.

Beyond this, many gaps must still be filled to provide adequate working conditions for psychologists acting in the prevention of AD and dementia in general, including the need for more adequate professional training in prevention.

In this context, neuropsychology plays a central role in both professional training and practice aimed at the prevention and mitigation of impairments associated with AD, by integrating knowledge of brain functioning, cognition, and behavior within clinical and educational contexts. In training, it provides theoretical and methodological foundations that enable the early identification of subtle cognitive deficits, thereby supporting preventive interventions and the development of cognitive stimulation strategies (Lezak et al., 2012; Strauss et al., 2006). In professional practice, systematic neuropsychological assessment allows for monitoring disease progression, facilitating differential diagnosis, and designing individualized rehabilitation plans, ultimately promoting the maintenance of functional abilities and quality of life (American Psychiatric Association, 2013; Petersen et al., 2014). Furthermore, evidence-based interventions, such as cognitive training and caregiver psychoeducation, have demonstrated potential in reducing the functional and emotional burden of AD, reinforcing the relevance of neuropsychology as a foundational axis in comprehensive care for cognitive aging (Belleville et al., 2006; Clare and Woods, 2004).

Additionally, new care models are critical to ensuring that new diagnostics and therapeutics are accessible to all, and if limited resources are directed only to the treatment of patients receiving disease-modifying therapies without simultaneously addressing care reform, well-established health disparities in access to dementia services will be exacerbated (Possin et al., 2024).

## 7 Limitations and future research perspectives

As a theoretical Perspective article, this manuscript is subject to several limitations that must be acknowledged. First, the proposed three-phase roadmap has not yet been empirically tested, and its real-world feasibility depends on structural and economic conditions that vary substantially across health systems. Economic barriers represent a central operational constraint as the cost of blood-based biomarker testing, specialized neuropsychological assessment, and psychologist salaries may be prohibitive for public health systems with constrained budgets, particularly in LMICs, where the shortage of psychologists integrated into multidisciplinary neurological and geriatric teams is already well documented (Kalaria et al., 2024). This shortage is compounded by unequal training opportunities and the near-total absence of standardized curricula preparing psychologists for pre-dementia preventive roles in these regions (Livingston et al., 2024).

Second, the feasibility of implementing Phase 2 and Phase 3 differs markedly between public and private healthcare systems. In predominantly private or out-of-pocket systems, access to blood-based biomarkers and specialized psychological follow-up may be restricted to individuals with sufficient purchasing power or private insurance, deepening existing health disparities; conversely, publicly funded systems, while more equitable in principle, often face long waiting times, staff shortages, and budget constraints that delay early psychological intervention (Schwarz et al., 2022). Reconciling these divergent realities within a single roadmap remains an open operational challenge that future health policy research must address directly.

Beyond these structural barriers, important knowledge gaps remain. There is still no consensus on standardized, manualized psychological intervention protocols specifically designed for individuals in the pre-dementia, at-risk stage, which limits the reproducibility and comparability of clinical practices across services and countries (Atri et al., 2025; Rostamzadeh et al., 2022). Longitudinal studies are needed to determine the long-term psychological and functional outcomes of structured risk disclosure and early psychoeducational intervention, as well as the cost-effectiveness of integrating psychologists into primary care teams for AD prevention. Future research should also prioritize the development and validation of culturally adapted intervention protocols for LMICs and underserved populations, alongside pragmatic trials evaluating telepsychology-based delivery models as a strategy to expand access under conditions of workforce scarcity.

To enable rigorous evaluation of this roadmap in future empirical studies, we propose a core set of candidate outcome indicators spanning psychological, cognitive, and health-system domains. At the individual level, relevant metrics include health-related quality of life (e.g., assessed via the EQ-5D or the Quality of Life in Alzheimer’s Disease scale), standardized measures of iatrogenic anxiety and depressive symptomatology following risk disclosure (e.g., the Hospital Anxiety and Depression Scale or the Geriatric Depression Scale), and indices of cognitive reserve and cognitive trajectory over time, such as performance on the PACC (Donohue et al., 2014). At the clinical-course level, time-to-event outcomes, particularly the rate and timing of conversion from an at-risk or preclinical state to mild cognitive impairment or clinical AD, would allow direct testing of whether structured psychological intervention delays disease progression. At the caregiver and family level, validated caregiver burden and anticipatory grief scales should be incorporated, consistent with the psychological impact on caregivers discussed above. Finally, at the health-system level, cost-effectiveness ratios, rates of psychologist integration into multidisciplinary teams, and equity-focused indicators (e.g., differential access across income strata or urban–rural settings) would allow policymakers to assess the roadmap’s scalability and fairness. Standardizing this set of metrics across future studies would substantially improve the comparability of findings and accelerate the translation of this proposal into evidence-based clinical practice.

## 8 Conclusion

This theoretical paper aimed to draw attention to the psychologist’s role and to the broader contribution of psychology in the prevention of AD in light of new diagnostic discoveries, considering the importance of the clinical approach, particularly from the perspective of the IWG. The IWG advocates for AD to be defined as a clinical-biological entity, with diagnosis made in the presence of an established clinical phenotype supported by pathophysiological biomarkers; biomarker investigations in cognitively normal individuals can be conducted in the context of non-diagnostic patient journeys aimed at evaluating the risk of future cognitive impairment, communicating it, and implementing risk reduction interventions (Dubois et al., 2024).

Psychology could reach a broader population in AD prevention, since environmental factors act on individuals throughout the lifespan as triggers of AD, and prevention must therefore be undertaken early. Fewer than 5% of AD and related dementia cases are due solely to genetic causes, and there is growing scientific consensus that these conditions arise from complex gene–environment interactions (Park et al., 2024). Risk reduction is a lifelong endeavor and is most effective when awareness and understanding of brain health begin at a young age, establishing good habits (Alzheimer’s Disease International, 2023). Theoretical models of neuropsychological rehabilitation hold that prevention involves applying strategies to enhance protective factors, such as cognitively stimulating and challenging activities, maintaining mood and emotional regulation, regular social interaction, adherence to healthier diets, and physical activity (Ramos-Galarza and Gaibor, 2025).

Government policies should be revised to integrate psychologists into multidisciplinary teams, and psychology schools must prepare students for the new challenges linked to the prevention of dementia, particularly AD. The 2024 Lancet Commission reported that addressing 14 modifiable risk factors across the lifespan could potentially prevent or delay up to 45% of dementia cases (Livingston et al., 2024). Dementia is shaped not only by genetics but also by socioeconomic, environmental, and lifestyle factors; poverty, limited healthcare access, and educational disparities heighten risk, while promoting healthier habits—including exercise, nutrition, and cognitive engagement—is essential (Mostert et al., 2025).

Special attention must be directed to at-risk patients identified as likely to develop AD through blood-based biomarker testing, and working groups should be created to develop strategies to improve the mental health of both patients and families. The IWG recommends that for cognitively normal individuals with positive biomarkers, appropriate support, counseling, and tailored risk reduction plans should be provided to help them process and manage this information (Dubois et al., 2024). Equally important is the continued development of strategies for the treatment of diagnosed AD. More research focusing specifically on the psychologist’s role is needed, considering the standardization of procedures, early prevention through cognitive and emotional education from childhood onward, and the promotion of healthier behaviors, particularly for those at higher genetic risk or living in adverse environmental conditions.

## Data Availability

The raw data supporting the conclusions of this article will be made available by the authors, without undue reservation.
